# Metformin attenuates lung fibrosis development via NOX4 suppression

**DOI:** 10.1186/s12931-016-0420-x

**Published:** 2016-08-30

**Authors:** Nahoko Sato, Naoki Takasaka, Masahiro Yoshida, Kazuya Tsubouchi, Shunsuke Minagawa, Jun Araya, Nayuta Saito, Yu Fujita, Yusuke Kurita, Kenji Kobayashi, Saburo Ito, Hiromichi Hara, Tsukasa Kadota, Haruhiko Yanagisawa, Mitsuo Hashimoto, Hirofumi Utsumi, Hiroshi Wakui, Jun Kojima, Takanori Numata, Yumi Kaneko, Makoto Odaka, Toshiaki Morikawa, Katsutoshi Nakayama, Hirotsugu Kohrogi, Kazuyoshi Kuwano

**Affiliations:** 1 Division of Respiratory Diseases; Department of Internal Medicine, Jikei University School of Medicine, 3-25-8 Nishi-shimbashi, Minato-ku, Tokyo, 105-8461 Japan; 2 Department of Respiratory Medicine, Faculty of Life Science, Kumamoto University, Kumamoto, Japan; 3 Research Institute for Diseases of the Chest, Graduate School of Medical Sciences, Kyushu University, Fukuoka, Japan; 4 Division of Chest Diseases; Department of Surgery, Jikei University School of Medicine, Tokyo, Japan

**Keywords:** IPF, Metformin, NOX4, ROS, TGF-β

## Abstract

**Background:**

Accumulation of profibrotic myofibroblasts in fibroblastic foci (FF) is a crucial process for development of fibrosis during idiopathic pulmonary fibrosis (IPF) pathogenesis, and transforming growth factor (TGF)-β plays a key regulatory role in myofibroblast differentiation. Reactive oxygen species (ROS) has been proposed to be involved in the mechanism for TGF-β-induced myofibroblast differentiation. Metformin is a biguanide antidiabetic medication and its pharmacological action is mediated through the activation of AMP-activated protein kinase (AMPK), which regulates not only energy homeostasis but also stress responses, including ROS. Therefore, we sought to investigate the inhibitory role of metformin in lung fibrosis development via modulating TGF-β signaling.

**Methods:**

TGF-β-induced myofibroblast differentiation in lung fibroblasts (LF) was used for in vitro models. The anti-fibrotic role of metfromin was examined in a bleomycin (BLM)-induced lung fibrosis model.

**Results:**

We found that TGF-β-induced myofibroblast differentiation was clearly inhibited by metformin treatment in LF. Metformin-mediated activation of AMPK was responsible for inhibiting TGF-β-induced NOX4 expression. NOX4 knockdown and N-acetylcysteine (NAC) treatment illustrated that NOX4-derived ROS generation was critical for TGF-β-induced SMAD phosphorylation and myofibroblast differentiation. BLM treatment induced development of lung fibrosis with concomitantly enhanced NOX4 expression and SMAD phosphorylation, which was efficiently inhibited by metformin. Increased NOX4 expression levels were also observed in FF of IPF lungs and LF isolated from IPF patients.

**Conclusions:**

These findings suggest that metformin can be a promising anti-fibrotic modality of treatment for IPF affected by TGF-β.

## Background

Accumulation of profibrotic myofibroblasts is a crucial process for fibrotic remodeling in idiopathic pulmonary fibrosis (IPF) [1]. Among a variety of profibrotic cytokines, transforming growth factor (TGF)-β has been widely implicated in IPF pathogenesis through regulating myofibroblast differentiation and proliferation [1]. Adenoviral transfer of TGF-β1 to rat lung induces prolonged severe interstitial fibrosis characterized by extensive deposition of extracellular matrix (ECM) proteins and accumulation of cells with a myofibroblast phenotype [2]. Integrin αvβ6-mediated physiological activation of TGF-β has been demonstrated to be involved in lung fibrosis development at least partly through epithelial-mesenchymal transition [3, 4]. With respect to a clinical implication, the concentrations of TGF-β1 in the bronchoalveolar lavage fluid (BALF) from IPF cases were significantly higher than those from control cases [5]. Hence, TGF-β is thought to play a crucial role in orchestrating fibrosis development during IPF pathogenesis and recent ongoing clinical trials have mainly focused on inhibition of fibrotic mechanisms, including TGF-β [6].

TGF-β-mediated biological activities are regulated via intracellular signaling pathways composed of canonical SMADs and SMAD-independent non-canonical pathways, including mitogen activated protein (MAP) kinases and phosphoinositide 3-kinase (PI3K) [7]. Reactive oxygen species (ROS) modulate TGF-β-induced cell signaling pathways via activating tyrosine kinases and inactivating protein tyrosine phosphatases, and NADPH oxidases (NOXes) are the major source of endogenous ROS production [8]. Among seven isoforms of NOXes, NOX4 has been shown to modulate TGF-β/SMAD-signaling via intracellular ROS production [8]. In comparison to other isoforms, NOX4 is unique in that it is constitutively active, thus its expression level is a major point of regulation [9]. Increased expression levels of NOX4 have been reported in IPF lung, including in myofibroblasts in fibroblastic foci (FF), suggesting the involvement of NOX4 in IPF pathogenesis through modulating TGF-β-induced myofibroblast differentiation [10, 11]. Recent papers also demonstrated potential therapeutic implications for a low-molecular weight NOX4 antagonist in prevention of bleomycin (BLM)-induced lung fibrosis [12]. Accordingly, NOX4 has been recognized to be a potential therapeutic target for IPF associated with enhanced TGF-β signaling.

Metformin is a commonly prescribed biguanide antidiabetic medication used to lower blood glucose in type II diabetes patients and also exhibits pleiotropic effects on cellular biology [13]. Metformin has been shown to reduce TGF-β-induced ECM protein production in fibroblasts derived from nasal polyps [14]. Furthermore, metformin prevented airway remodeling in mouse models of bronchial asthma, suggesting a potential anti-fibrotic property [15]. Accordingly, recent papers have demonstrated metformin-mediated attenuation of bleomycin (BLM) and gefitinib-induced lung fibrosis through regulation of TGF-β signaling [16]. Pharmacological action of metformin is mediated via the phosphorylation of AMP-activated protein kinase (AMPK) [17], and AMPK regulates not only intracellular energy balance via lipid and glucose metabolism but also a wide array of cell functions [18]. AMPK activation by metformin was responsible for inhibiting TGF-β-induced collagen production in mouse renal fibroblasts [19]. Furthermore, AMPK has been demonstrated to negatively regulate NOX4 expression in glomerular epithelial cells [20]. We therefore examined the inhibitory mechanisms of metformin in TGF-β-induced myofibroblast differentiation of lung fibroblasts (LF), and also evaluated the anti-fibrotic role of metformin by using bleomycin (BLM)-induced lung fibrosis mouse models in relation to AMPK activation and NOX4 suppression.

## Methods

### Cell culture, antibodies, and reagents

Normal lung tissues were obtained from pneumonectomy and lobectomy specimens from primary lung cancer. Informed consent was obtained from all surgical participants as part of an approved ongoing research protocol by the ethical committee of Jikei University School of Medicine {#20-153 (5443)}. Lung fibroblasts (LF) were isolated and characterized as previously described [21]. Briefly, LF outgrown from lung fragments were cultured in fibroblast growth media (DMEM with 10 % FCS and penicillin-streptomycin). LF were serially passaged and used for experiments until passage 6. LF demonstrated >95 % positive staining with anti-vimentin antibodies, and <5 % positive staining with the anti-cytokeratin antibody (Data not shown). Antibodies used were rabbit anti-AMPKα (Cell Signaling Technology, # 2532), rabbit anti-phospho-AMPKα (T172) (Cell Signaling Technology, # 2535), rabbit anti-NOX4 (Novus, # NB110-58849), goat anti-type I collagen (Southern Biotech, # 1310-01), mouse anti-α smooth muscle actin (Sigma-Aldrich, # A2547), rabbit anti-SMAD2 (Cell Signaling Technology, # 3122), rabbit anti-SMAD3 (Cell Signaling Technology, # 9513), rabbit anti-phospho-SMAD2 (Cell Signaling Technology, # 3101), rabbit anti-phospho-SMAD3 (Cell Signaling Technology, # 8769), rabbit anti- phospho-SMAD3 (phospho S423 + S425) (Abcam, # 52903), and mouse anti-β-actin (Sigma-Aldrich, # A5316). Metformin was provided from Sumitomo Dainippon Pharma Co, Tokyo, Japan. Recombinant human TGF-β1 (R&D Systems, # 100-B), N-acetylcysteine (NAC) (Wako, # 017-05131), CM-H2DCFDA (Life Technologies, # C6827), and bleomycin (Nippon Kayaku Co., Tokyo, Japan) were purchased.

### siRNA and transfection

Small interfering RNA (siRNA) targeting AMPK (Applied Biosystems Life Technologies, # 4392420, ID:S100 and S102), NOX4 (QIAGEN, Hs_NOX4_6 FlexiTube siRNA, # SI02642507 and Applied Biosystems Life Technologies, # 4392420, ID:27013), and negative control siRNAs (Applied Biosystems Life Technologies, # AM4635 and # AM4641) were purchased. Specific knockdowns of AMPK, and NOX4 were validated using two different siRNA, respectively. Transfections of LF were performed using the Neon® Transfection System (Invitrogen Life Technologies, # MPK5000), using matched optimized transfection kits (Invitrogen Life Technologies, # MPK10096).

### RNA isolation, polymerase chain reaction

RNA isolation, reverse transcription and Real-Time PCR were performed using the SYBR green method as previously described [21]. The primers used were NOX4 sense primer, 5’- CAGATGTTGGGGCTAGGATTG -3’; NOX4 antisense primer, 5’- GAGTGTTCGGCACATGGGTA -3’; β-actin sense primer 5’-CATGTACGTTGCTATCCAGGC -3’ β-actin antisense primer 5’-CTCCTTAATGTCACGCACGAT -3’. These primer sets yielded PCR products of 96 bp and 250 bp for NOX4 and β-actin respectively. Primer sequences were from Primer Bank (http://pga.mgh.harvard.edu/primerbank.)

### Measurement of ROS production

LF, at a density of 5 × 10^3^ per well, were seeded in a 96-well microplate (Thermo Fisher Scientific, # 237105). CM-H2DCFDA was used to measure total cellular ROS according to the manufacturer’s instructions. After incubation with CM-H2DCFDA (10 μM) for 30 min at 37 °C, fluorescence of DCF was measured at an excitation wavelength of 485 nm and an emission wavelength of 535 nm by a fluorescence microplate reader (Infinite F 200) (Tecan Japan, Kanagawa, Japan).

### Western blotting

LF grown on 6-well culture plates were lysed in RIPA buffer (Thermo Fisher Scientific, catalog # 89900) with protease inhibitor cocktail (Roche Diagnostics, # 11697498001) and 1 mM sodium orthovanadate, or lysed with Laemmli sample buffer. Western blotting was performed as previously described [21, 22]. For each experiment, equal amounts of total protein were resolved by 7.5‐10 % SDS/PAGE. After SDS/PAGE, proteins were transferred to polyvinylidene difluoride (PVDF) membrane (Millipore, # ISEQ00010), and incubation with specific primary antibody was performed for 1 h at 37 °C, or 24 h at 4 °C. After washing several times with PBST, the membrane was incubated with Anti-rabbit IgG, HRP-linked secondary antibody (Cell Signaling Technology, # 7074), Anti-mouse IgG, HRP-linked secondary antibody, # 7076) or Anti-goat IgG (H + I), HRP-linked secondary antibody (BETHYL, #A50-100P) followed by chemiluminescence detection (Thermo scientific, # 34080, and BIO-RAD, # 1705061) with the LAS-4000 UVmini system (Fujifilm, Tokyo, Japan) and ChemiDocTM Touch Imaging System (BIO-RAD, California, USA).

### Mouse models

C57BL/6J mice were purchased (CLEA Japan INC, Tokyo, Japan) and were maintained in the animal facility at the Jikei University School of Medicine. All experimental procedures are approved by the Jikei University School of Medicine Animal Care Committee (#25031). A dose of 3 U/kg bleomycin (Nippon Kayaku Co., Tokyo, Japan) was intratracheally administered in 50 μL saline using MicroSprayer™ Aerosolizer and a high pressure syringe (PennCentury, Philadelphia, PA). Intraperitoneal dose of metformin (300 mg/kg) were given from day 7 to day 20. On the 21th day the lungs were removed. The lungs were fixed overnight in 10 % buffered formalin, embedded in paraffin, and the sections are stained with hematoxylin & eosin (HE).

### Masson’s trichrome staining and immunohistochemistry

To evaluate the changes of collagen deposition in lungs, Masson’s trichrome staining was performed as previously described [22]. Immunohistochemical staining was performed as previously described with minor modifications on the paraffin-embedded lung tissues [21, 22]. N-Histofine MOUSESTAIN KIT (Nichirei Biosciences Inc., # 414321) was used for immunohistochemical staining of mouse lung sections.

### Sircol soluble collagen assay

For quantitatively measuring collagen in mouse left lungs, the Sircol soluble collagen assay was performed according to the manufacturer’s instructions (Biocolor Life Science Assay, # S100).

### Statistics

Data are shown as the average (±SEM) taken from at least three independent experiments. Student’s *t*-test was used for comparison of two data sets, analysis of variance for multiple data sets. Tukey’s or Dunn’s test were used for parametric and nonparametric data, respectively, to find where the difference lay. Significance was defined as *p* < 0.05. Statistical software used was Prism v.5 (GraphPad Software, Inc., San Diego, CA).

## Results

### Metformin inhibits TGF-β-induced myofibroblast differentiation via AMPK activation in LF

**Fig. 1:**
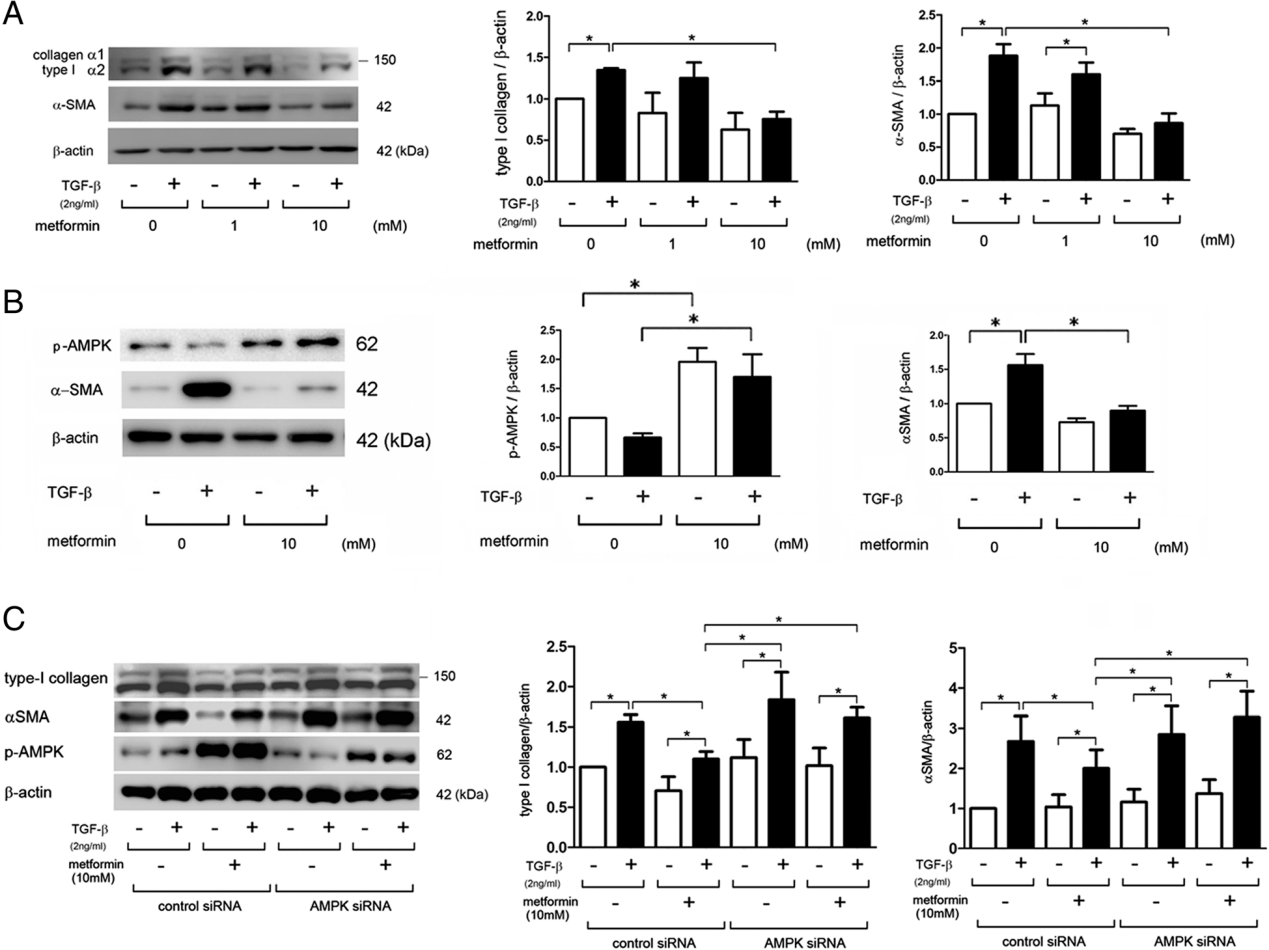
Metformin inhibits myofibroblast differentiation through AMPK activation in LF. **a** Western blotting (WB) using anti-type I collagen, anti-α-smooth muscle actin (SMA), and anti-β-actin of cell lysates from control (lane 1, 2), metformin (1 mM) (lane 3, 4), and metformin (10 mM) (lane 5, 6) treated LF. Metformin treatment was started 1 h before TGF-β (2 ng/ml) stimulation and protein samples were collected after 24 h treatment with TGF-β. In the right panels are the average (±SEM) taken from three independent experiments shown as relative expression. Open bar is control and filled bar is TGF-β treated. *p < 0.05. **b** WB using anti-phospho-AMPK, anti-αSMA, and anti-β-actin of cell lysates from control (lane 1, 2) and metformin (10 mM) (lane 3, 4) treated LF. Metformin treatment was started 1 h before TGF-β (2 ng/ml) stimulation and protein samples were collected after 24 h treatment with TGF-β. In the right panels are the average (±SEM) taken from three independent experiments shown as relative expression. Open bar is control and filled bar is TGF-β treated. *p < 0.05. **c** WB using anti-type I collagen, anti-αSMA, anti-phospho-AMPK, and anti-β-actin of cell lysates from control siRNA (lane 1, 2, 3, 4) and AMPK siRNA (lane 5, 6, 7, 8) transfected LF. Metformin treatment was started 48 h post transfection and 1 h before TGF-β (2 ng/ml) stimulation. Protein samples were collected after 24 h treatment with TGF-β. The right panels show the average (±SEM) of type I collagen and αSMA relative expression, which were taken from five to six independent experiments, respectively. Open bar is control and filled bar is TGF-β treated. *p < 0.05

TGF-β induced myofibroblast differentiation is shown by an increase in type I collagen and αSMA expression levels in LF (Fig. 1a, b, c). Metformin suppressed myofibroblast differentiation in a dose dependent manner and significant reduction was observed at concentrations of 10 mM (Fig. 1a). Hence, a metformin concentration of 10 mM was chosen for further analysis of cell culturing models. The pharmacological action of metformin is mainly mediated through activation of AMPK [17]. Metformin-induced AMPK activation was confirmed by detecting the phosphorylated form of AMPK with concomitant suppression of αSMA expression levels (Fig. 1b).

To elucidate the involvement of AMPK activation in regulation of myofibroblast differentiation by metformin, we employed siRNA-mediated AMPK knockdown. AMPK knockdown clearly reduced the amount of phosphorylation of AMPK following metformin treatment. In line with recent findings [16], inhibition of myofibroblast differentiation by metformin was clearly abrogated by AMPK knockdown, indicating that AMPK activation is involved in this inhibition (Fig. 1c).

### NOX4 is involved in metformin-mediated inhibition of myofibroblast differentiation in LF

**Fig. 2:**
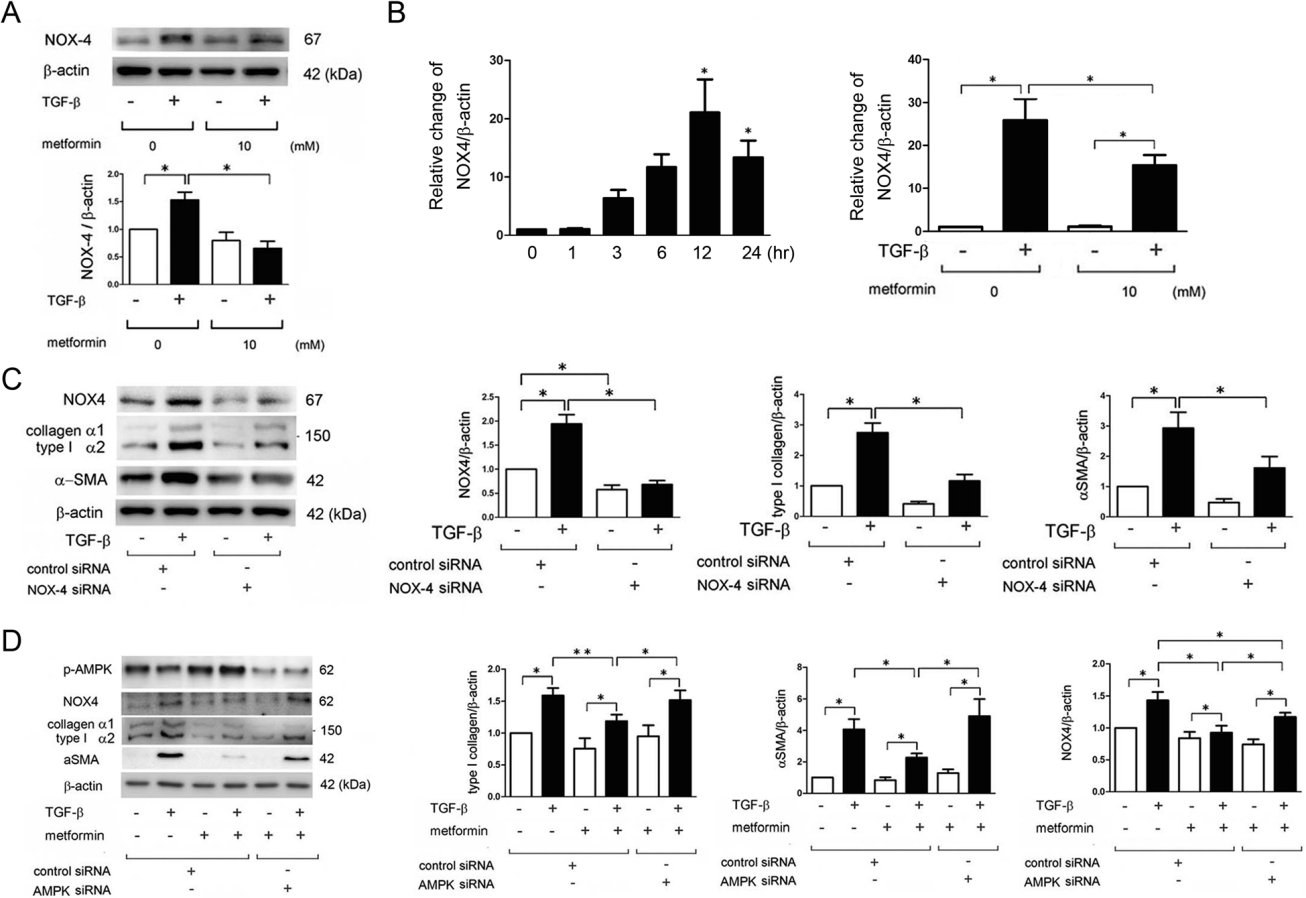
Metformin-mediated AMPK activation is involved in suppression of TGF-β-induced NOX4 expression in LF. **a** WB using anti-NOX4, and anti-β-actin of cell lysates from control (lane 1, 2) and metformin (lane 3, 4) treated LF. Metformin treatment was started 1 h before TGF-β (2 ng/ml) stimulation and protein samples were collected after 24 h treatment with TGF-β. Lower panel is the average (±SEM) taken from three independent experiments shown as relative expression. Open bar is control and filled bar is metformin treated. *p < 0.05. **b** Left panel: LF were treated with TGF-β and mRNA samples were collected at indicated time points (*n* = 9). *p < 0.05. Right panel: LF were treated with TGF-β in the presence or absence of metformin (10 mM) and mRNA samples were collected after 12 h treatment with TGF-β (*n* = 6). Open bar is control and filled bar is metformin treated. Real time-PCR was performed using primers to NOX4 or β-actin, as a control. NOX4 expression was normalized to β-actin. Shown is the fold increase (±SEM) relative to control treated cells. *p < 0.05. **c** WB using anti-NOX4, anti-type I collagen, anti-α-smooth muscle actin (SMA) and anti-β-actin of cell lysates from control siRNA (lane 1, 2) and NOX4 siRNA (lane 3, 4) transfected LF. TGF-β (2 ng/ml) treatment was started 48 h post transfection. Protein samples were collected after 24 h treatment with TGF-β. In the right panels are the average (±SEM) taken from four independent experiments shown as relative expression. Open bar is control and filled bar is TGF-β treated. *p < 0.05. **d** WB using anti-phospho-AMPK, anti-NOX4, anti-type I collagen, anti-αSMA, and anti-β-actin of cell lysates from control siRNA (lane 1, 2, 3, 4) and AMPK siRNA (lane 5, 6) transfected LF. Metformin treatment was started 48 h post transfection and 1 h before TGF-β (2 ng/ml) stimulation, and protein samples were collected after 24 h treatment with TGF-β. In the right panels are the average (±SEM) taken from five independent experiments shown as relative expression. Open bar is control and filled bar is TGF-β treated. *p < 0.05

Recent papers demonstrated a pivotal role for NOX4 in TGF-β signaling and myofibroblast differentiation [11]. To elucidate the participation of NOX4 in metformin-mediated regulation of myofibroblast differentiation, the changes in NOX4 expression levels following TGF-β treatment were evaluated in the presence or absence of metformin. TGF-β significantly enhanced NOX4 expression at the protein level, which was significantly suppressed by metformin (Fig. 2a). TGF-β also increased NOX4 expression at the mRNA level, which peaked at 12 hr post-treatment (Fig. 2b left panel). Metformin treatment subsequently showed efficient inhibition of TGF-β-induced NOX4 mRNA (Fig. 2b right panel). NOX4 siRNA was employed and efficient knockdown was confirmed by western blotting (Fig. 2c). TGF-β-induced myofibroblast differentiation was clearly inhibited by NOX4 knockdown (Fig. 2c). To confirm the association between AMPK and NOX4, the changes of expression levels of NOX4 following metformin treatment were examined in the setting of AMPK knockdown. Metformin-mediated attenuation of NOX4 and αSMA expression during TGF-β treatment was efficiently restored by AMPK knockdown (Fig. 2d).

**Fig. 3:**
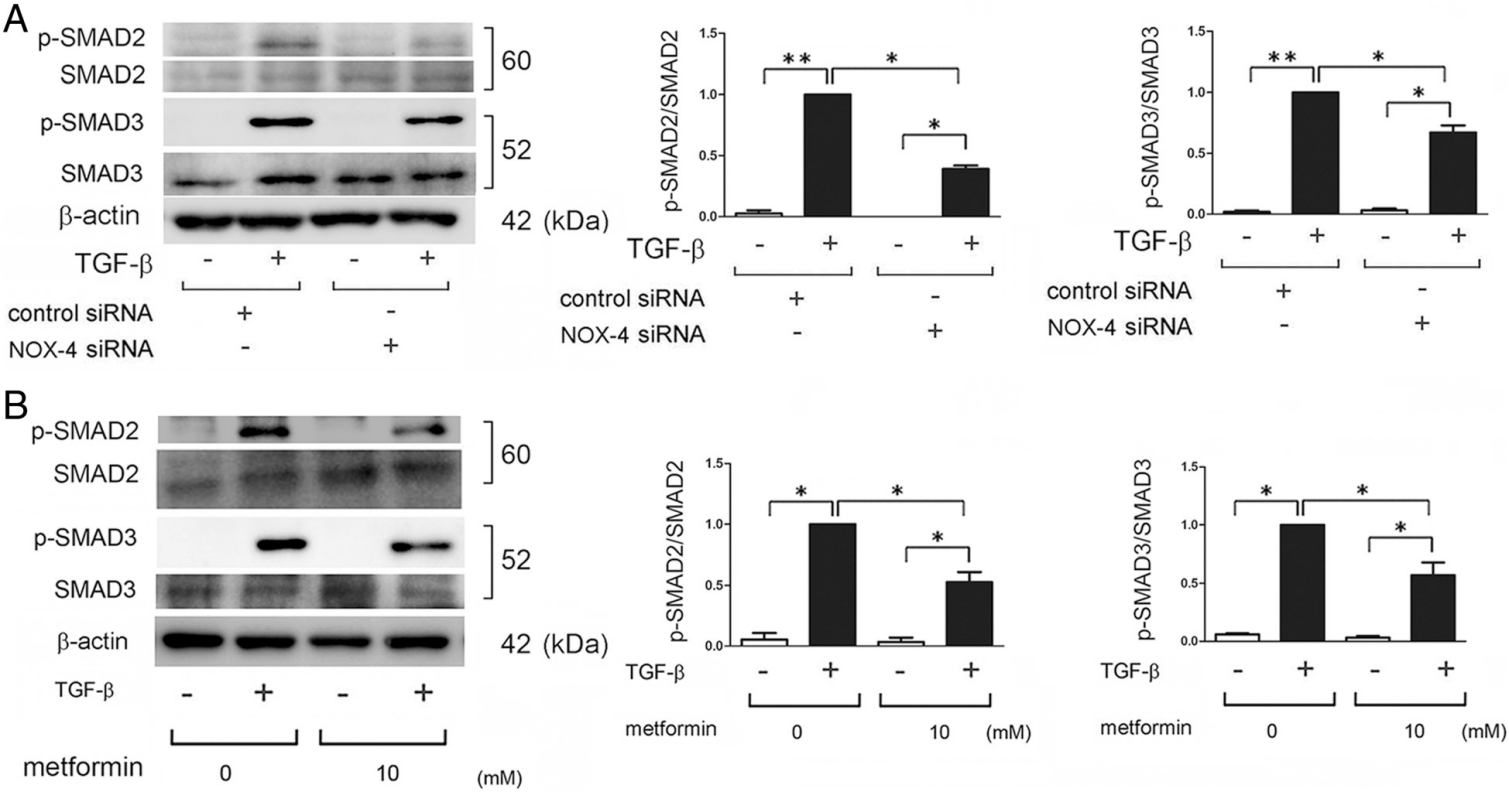
Metformin and NOX4 regulate SMAD phosphorylation in LF. **a** WB using anti-phospho-SMAD2, anti-SMAD2, anti-phospho-SMAD3, anti-SMAD3, and anti-β-actin of cell lysates from control siRNA (lane 1, 2) and NOX4 siRNA (lane 3, 4) transfected LF. TGF-β (2 ng/ml) treatment was started 48 h post transfection. Protein samples were collected after 30 min treatment with TGF-β. In the right panels are the average (±SEM) taken from three independent experiments shown as relative expression. Open bar is control and filled bar is TGF-β treated. *p < 0.05. **b** WB using anti-phospho-SMAD2, anti-SMAD2, anti-phospho-SMAD3, anti-SMAD3, and anti-β-actin of cell lysates from control (lane 1, 2) and metformin (10 mM) (lane 3, 4) treated LF. Metformin treatment was started 1 h before TGF-β (2 ng/ml) stimulation and protein samples were collected after 30 min treatment with TGF-β. In the right panels are the average (±SEM) taken from three independent experiments shown as relative expression. Open bar is control and filled bar is TGF-β treated. *p < 0.05

NOX4 has been implicated as both an upstream and a downstream mediator of TGF-β-mediated SMAD signaling [8]. NOX4 knockdown attenuated phosphorylation of SMAD2 and SMAD3 30 min after TGF-β treatment (Fig. 3a). In line with the NOX4 knockdown experiments, metformin significantly suppressed both SMAD2 and SMAD3 phosphorylation 30 min after TGF-β treatment (Fig. 3b).

### NOX4-mediated ROS production is responsible for TGF-β-induced myofibroblast differentiation in LF

**Fig. 4:**
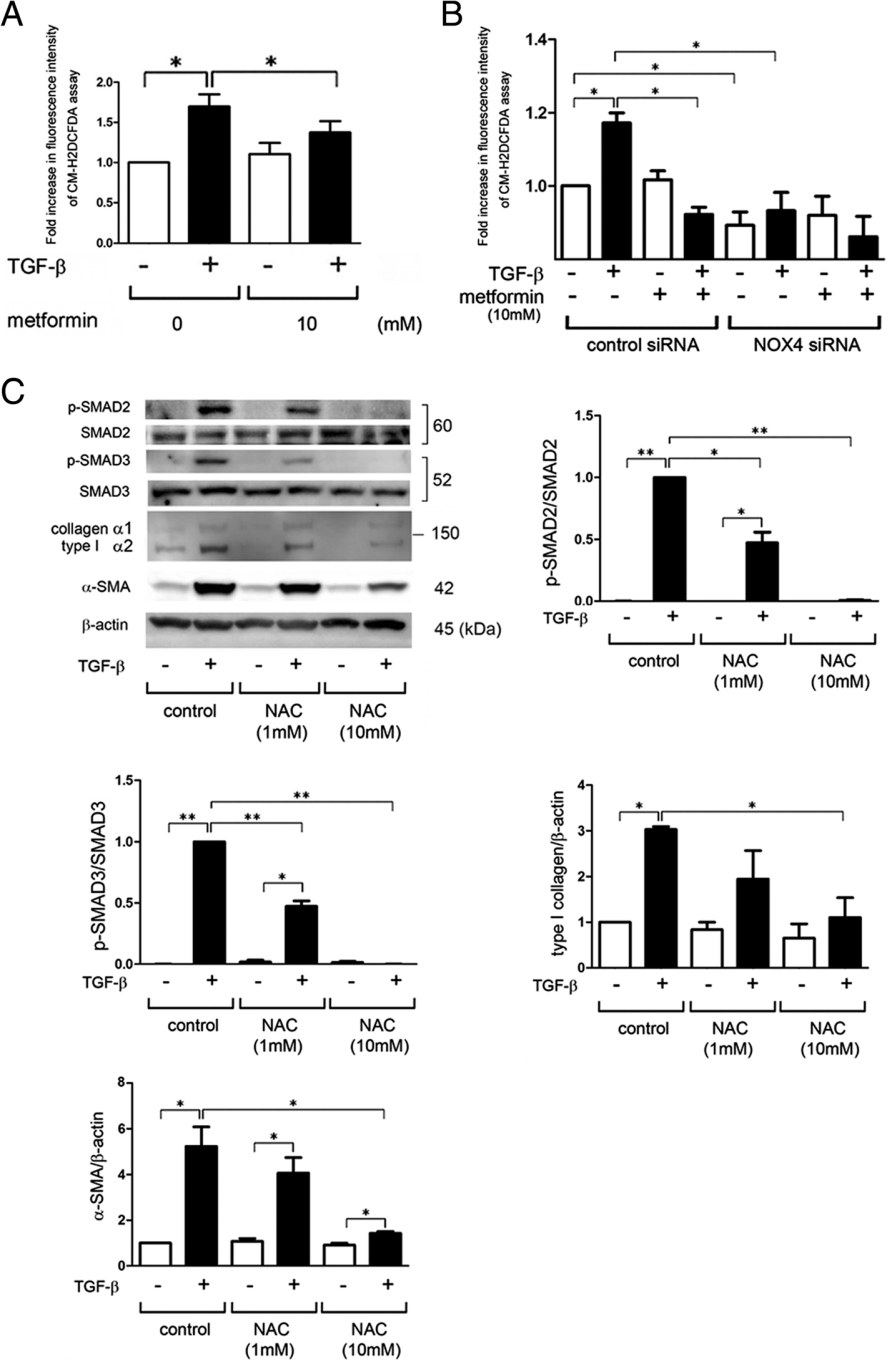
NOX4-mediated ROS is involved in the mechanisms for SMAD phospholylation and myofibroblast differentiation in LF. **a** Fluorescence intensity of CM-H2DCFDA staining for intracellular ROS production. After 24 h treatment with TGF-β, incubation with CM-H2DCFDA (10 μM) was performed for 30 min, fluorescence of DCF was measured by a fluorescence microplate reader. The fluorescence level in the control treated cells in the absence of metformin was designated as 1.0. Shown panels are the average (±SEM) taken from three independent experiments. *p < 0.05. **b** Fluorescence intensity of CM-H2DCFDA staining for intracellular ROS production. Metformin treatment was started 48 h post-siRNA transfection and 1 h before TGF-β (2 ng/ml) stimulation. After 30 min incubation with CM-H2DCFDA, fluorescence of DCF was measured by a fluorescence microplate reader. The fluorescence level in the control siRNA transfected cells without TGF-β and metformin treatment was designated as 1.0. Shown panels are the average (±SEM) taken from six independent experiments. *p < 0.05. **c** WB using anti-phospho-SMAD2, anti-SMAD2, anti-phospho-SMAD3, anti-SMAD3, anti-type I collagen, anti-αSMA, and anti-β-actin of cell lysates from control (lane 1, 2), NAC (1 mM) (lane 3, 4), and NAC (10 mM) (lane 5, 6) treated LF. NAC treatment was started 1 h before TGF-β (2 ng/ml) stimulation and protein samples were collected after 24 h treatment for type I collagen and anti-αSMA WB, but 30 min for SMAD WB. Shown panels are the average (±SEM) taken from three independent experiments shown as relative expression. Open bar is control and filled bar is TGF-β treated. *p < 0.05 and **p < 0.001

NOX4-mediated hydrogen peroxide (H_2_O_2_) production of redox pathway modulation has been implicated in regulating TGF-β signaling [8], hence intracellular ROS production was examined by means of the CM-H2DCFDA assay. TGF-β treatment induced ROS production, which was significantly reduced by metformin treatment (Fig. 4a). Knockdown experiments confirmed that NOX4 is mainly responsible for TGF-β-induced ROS production (Fig. 4b). No significant additional inhibition of ROS production was observed by metformin treatment in NOX4 knockdown LF (Fig. 4b). Involvement of TGF-β-induced ROS production in SMAD signaling and myofibroblast differentiation was also examined by using N-acetylcysteine (NAC), a representative intracellular antioxidant. NAC treatment significantly suppressed TGF-β-induced SMAD2/3 phosphorylation and myofibroblast differentiation at the concentration of 10 mM (Fig. 4c).

### Metformin attenuates bleomycin-induced lung fibrosis development in mice

**Fig. 5:**
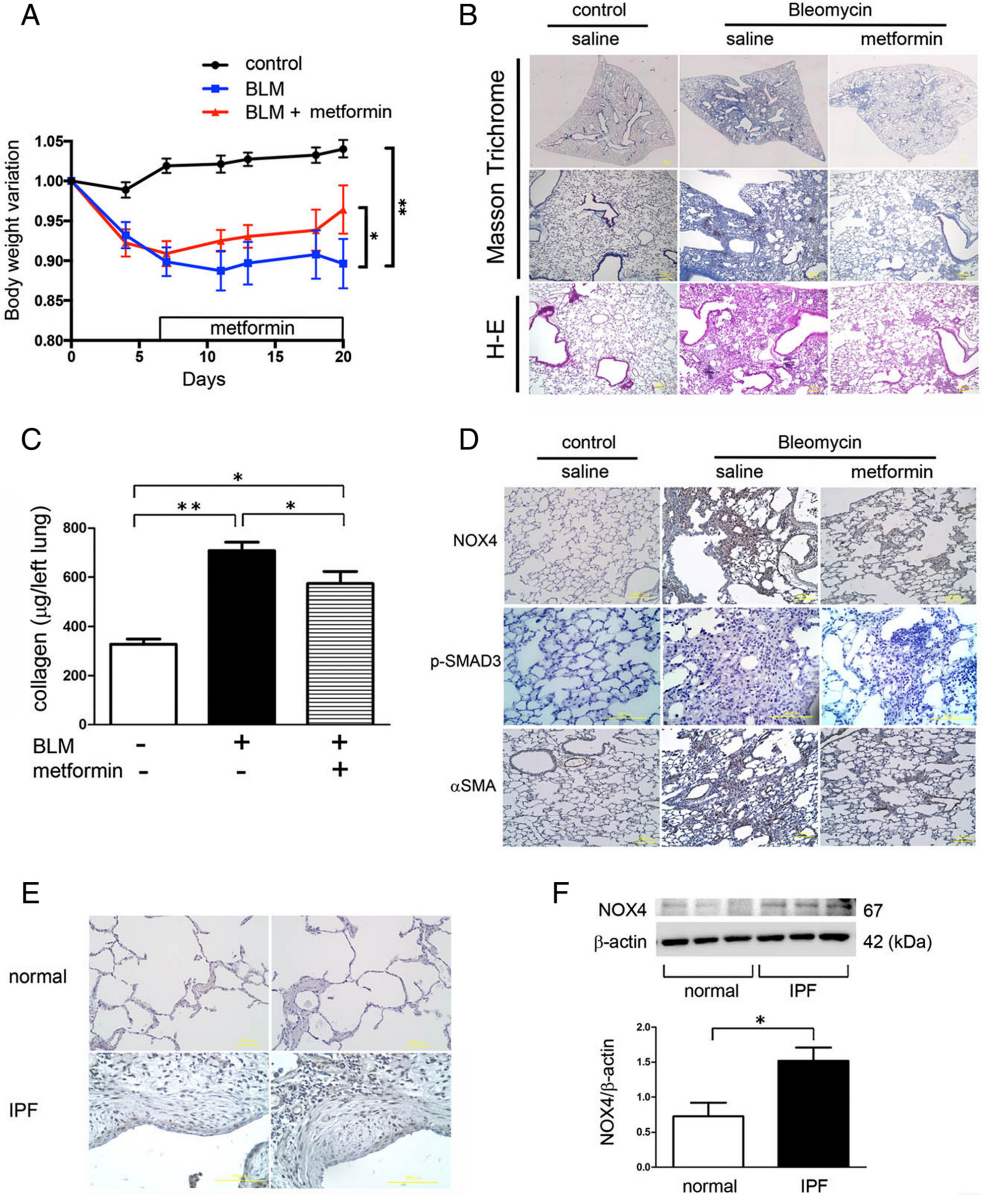
Effect of metformin on bleomycin-induced lung fibrosis development in mice. **a** Body Wight (BW) changes after BLM treatment. BW at day 0 before treatment was designated as 1.0. *p < 0.05. **b** Photomicrographs of Masson trichrome and Hematoxylin-Eosin staining of mouse lungs at day 21. Upper panels are low magnification view of Masson trichrome staining. Original magnification × 40. Middle panels are High magnification view of Masson trichrome staining. Original magnification × 100. Lower panels are high magnification view of Hematoxylin-Eosin staining. Original magnification × 100. **c** Shown in the panel is the average (±SEM) soluble collagen measurement from Sircol assay using control (*n* = 13), BLM-treated (*n* = 18), and BLM-treated with subsequent metformin injection mouse lungs (*n* = 15) at day 21. Open bar is control, filled bar is BLM-treated, and horizontal crosshatched bar is BLM-treated with subsequent metformin injection. *p < 0.05. **d** Immunohistochemical staining of NOX4, p-SMAD3, αSMA in mouse lungs at day 21. Upper panels are high magnification view of NOX4 staining. Original magnification × 200. Middle panels are High magnification view of p-SMAD3 staining. Original magnification × 400. Lower panels are high magnification view of αSMA staining. Original magnification × 200. Bar = 100 μm **e** Immunohistochemical staining of NOX4 in human lungs. Upper panels are high magnification view of normal lungs. Original magnification × 200. Lower panels are High magnification view of IPF lungs. Original magnification × 400. Bar = 100 μm **f** WB using anti-NOX4, and anti-β-actin of cell lysates from normal LF (lane 1, 2, 3) and IPF LF (lane 4, 5, 6). Lower panel is the average (±SEM) taken from three patients shown as relative expression. Open bar is normal LF and filled bar is IPF LF. *p < 0.05

Next, mouse models of BLM-induced lung fibrosis were used to examine the anti-fibrotic action of metformin via NOX4 modulation. To show a possible clinical relevance for metformin in treatment of IPF, intraperitoneal metformin injection was initiated on day 7 following BLM treatment. In general, day 7 is considered to be the beginning of the fibrotic phase with concomitant resolution of acute inflammatory reaction. Compared with control treated mice, BLM treated mice showed significant body weight loss, which was markedly recovered during metformin treatment (Fig. 5a). Metformin treatment clearly and significantly reduced lung fibrosis development at day 21 by means of Masson trichrome staining and Sircol collagen assay, respectively (Fig. 5b, c).

To elucidate participation of TGF-β signaling through the NOX4-SMAD axis in the BLM-induced lung fibrosis and in attenuation of fibrosis by metformin, lung samples at day 21 were examined by immunohistochemistry. Compared with control treated lungs, increased NOX4, p-SMAD3, and αSMA expression were clearly observed in fibrotic lesions in BLM-treated lungs (Fig. 5d). Consistent with the results of in vitro experiments, metformin clearly suppressed NOX4, p-SMAD3, and αSMA expression levels in BLM-treated lungs (Fig. 5d). In line with recent reports, clinical implications for NOX4 in IPF pathogenesis for Japanese patients were further confirmed by showing positive NOX4 staining in FF fibroblasts (Fig. 5e). In comparison to LF from normal lungs, LF isolated from IPF lungs also showed increased NOX4 expression levels (Fig. 5f).

## Discussion

**Fig. 6:**
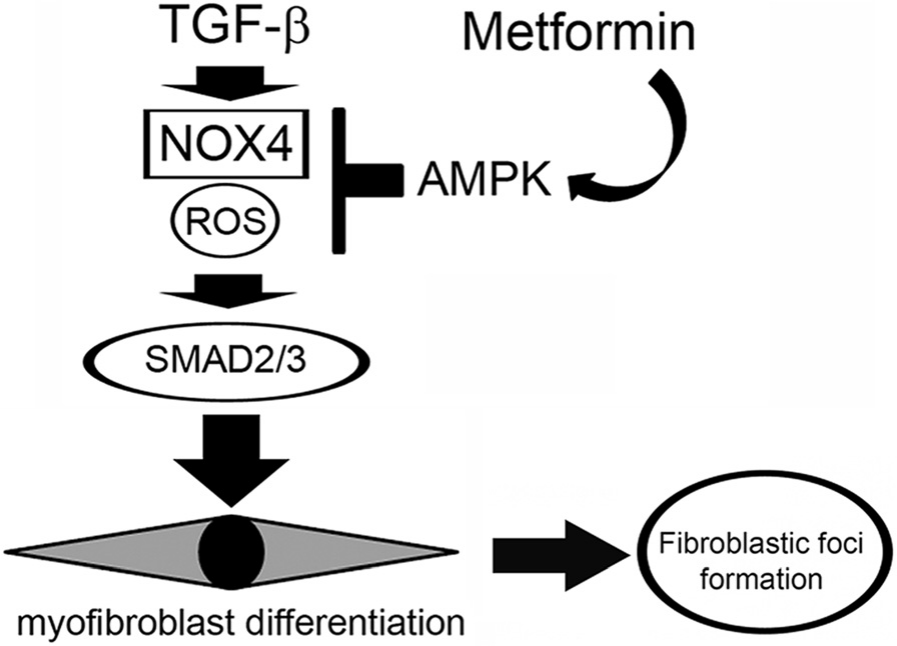
Hypothetical model of metformin-mediated inhibition of myofibroblast differentiation. Metformin-mediated AMPK activation is responsible for inhibiting NOX4 expression and ROS production, which is at least partly involved in the mechanisms for attenuation of TGF-β-induced SMAD phosphorylation and myofibroblast differentiation in relation to fibroblastic foci formation in IPF pathogenesis

In the present study, we demonstrate that metformin-mediated AMPK activation is involved in the mechanisms for attenuation of TGF-β-induced myofibroblast differentiation in LF through inhibiting NOX4 expression (Fig. 6). Metformin regulates TGF-β-induced NOX4 expression at the mRNA level and NOX4 is responsible for TGF-β-induced endogenous ROS production in LF. Metformin treatment with concomitant NOX4 knockdown indicates that NOX4 is mainly involved in the mechanisms for metformin-mediated ROS inhibition during TGF-β treatment (Fig. 4b). Metformin reduces the expression levels of NOX4, SMAD phosphorylation, and αSMA with concomitant attenuation of lung fibrosis in BLM treatment, suggesting that the anti-fibrotic mechanism of metformin is mainly attributable to inhibition of TGF-β-mediated myofibroblast differentiation. In line with recent findings, increased NOX4 expression levels are also observed in FF fibroblasts of IPF lungs and LF isolated from IPF lungs [10, 11]. Accordingly we speculate that metformin regulation of NOX4 expression can be a promising anti-fibrotic modality of treatment for fibrotic lung disorders affected by TGF-β. Although recent papers also showed an anti-fibrotic role for metformin in BLM-induced lung fibrosis models [16], efficient inhibition of BLM-induced lung fibrosis by metformin administration during the fibrotic phase in the present study further sheds light on the potential clinical usefulness of metformin for the treatment of IPF with ongoing fibrotic process.

Metformin exhibits pleiotropic mechanisms for cell protection, mainly through AMPK activation. In addition to energy metabolism, AMPK has been shown to be involved in the regulation of various cellular processes, including proliferation, mitochondrial integrity, inflammatory response, ER stress, and oxidative stress [18]. AMPK activation is recognized to have potential beneficial effects not only on improving metabolic disorders but also on preventing organ dysfunction during fibrosis development, including pulmonary diseases [23]. AMPK activation has been implicated in metformin-mediated effectiveness against a variety of lung pathologies, including lung cancer, bronchial asthma, tuberculosis, cigarette smoke-induced lung damages, ventilator-induced lung injury, and lipopolysaccharide (LPS)-induced lung injury [13, 15, 24–27]. Furthermore, a recent paper demonstrated that TGF-β-induced myofibroblast differentiation and BLM-induced lung fibrosis were efficiently suppressed by metformin-mediated AMPK activation [16]. In our present study, we have further elucidated that AMPK-mediated NOX4 suppression in particular is involved in metformin’s anti-fibrotic mechanisms.

NOX4 has been implicated as both an upstream and downstream mediator in TGF-β signaling [8]. In line with the NOX4 knockdown experiment, we showed that metformin significantly suppressed SMAD phosphorylation (Fig. 3) and ROS production at 30 min after TGF-β treatment (data not shown), suggesting that metformin-mediated ROS suppressing mechanisms, including NOX4 regulation, may participate in the inhibition of SMAD phosphorylation during TGF-β treatment. We have also treated LF with hydrogen peroxide (100 μM) in the presence or absence of TGF-β. However no effect on SMAD phosphorylation was demonstrated by hydrogen peroxide (data not shown), indicating not only the different role between NOX4-mediated ROS and extrinsic ROS but also permissive role of ROS in regulating cell signaling by TGF-β. TGF-β-induced NOX4 expression is also dependent on SMAD signaling, suggesting the existence of a self-amplifying loop of TGF-β signaling and NOX4 expression [8]. Intriguingly, recent papers showed that NOX4 is essential for not only myofibroblast differentiation but also subsequent phenotypic alterations to apoptosis resistance by accelerating cellular senescence in LF, which is associated with prolonged ECM production during IPF pathogenesis [11, 28]. Along with regulation of the myofibroblast phenotype in LF, NOX4 has also been implicated in the regulation of TGF-β-induced apoptosis in epithelial cells. In the case of NOX4 deficiency, due to loss of intrinsic ROS generation, TGF-β failed to induce apoptosis in alveolar epithelial cells (AEC) [10, 29]. Increase in NOX4 expression levels was observed not only in LF of actively fibrosing areas but also injured epithelial cells in IPF lungs [12, 28]. Hence, apoptosis inhibition in AEC by NOX4 suppression can also be a beneficial part of metformin treatment during IPF. siRNA-meditated NOX4 knockdown and low-molecular-weight NOX4 antagonist have been shown to efficiently attenuate BLM-induced lung fibrosis [12], further supporting the notion that metformin-mediated NOX4 suppression can be a reasonable and promising IPF treatment.

Due to the relative paucity of inflammatory cell infiltration as well as the failure of anti-inflammatory and immunosuppressive modality of treatments, the aberrant wound healing process of excessive myofibroblast accumulation has been recognized to be an essential pathology for IPF development [30]. Recently available medical treatments showing significant reduction in the rate of decline of forced vital capacity are mainly mediated through anti-fibrotic mechanisms [31, 32]. Furthermore, the majority of ongoing clinical trials for IPF treatment are based on the mechanisms of fibrogenesis, including TGF-β [6]. In general, discovery and development of new drugs are a difficult and time-consuming process with unpredictable adverse events. Drug repositioning is a recently proposed new drug discovery strategy whereby a library of approved drugs is screened for new indications [33]. The advantages of drug repositioning are decreased risks for unexpected adverse effects and simplified clinical trials. Metformin is widely used for type II diabetes patients in clinical settings with acceptable adverse events [34]. Hence, our findings of an anti-fibrotic property of metformin indicate that metformin can, through drug repositioning, be an alternative approach for IPF treatment. In comparison to clinically achievable plasma metformin concentrations, we used higher concentrations of metformin in in vitro experiments. Previous reports showing anti-fibrotic and anti-inflammatory properties also selected similar concentrations of metformin as used in our experiments [15, 16, 35], suggesting that a higher concentration is necessary to see the efficacy in in vitro conditions. However, mice were treated with 300 mg/kg of metformin, which is estimated to be comparable to a metformin dose of around 1500 mg/day for a 60 kg human [15]. Although we selected intraperitoneal administration in our mouse models, bioavailability of oral administration of metformin is calculated as 50 to 60 % in humans. Hence we speculate that the currently used maximum metformin dose for diabetes treatment (2250 mg/day in Japan) might be sufficient to see the anti-fibrotic properties of metformin treatment during IPF, which should be evaluated in future studies.

## Conclusions

In summary, we elucidated that metformin, an AMPK activator, attenuates lung fibrosis development by inhibiting TGF-β signaling through NOX4 suppression. We consider metformin to be a promising candidate agent for an anti-fibrotic modality of treatment for IPF patients.

## Abbreviations

AEC: Alveolar epithelial cells
AMPK: AMP-activated protein kinase
BALF: Bronchoalveolar lavage fluid
BLM: Bleomycin
BW: Body weight
CM-H2DCFDA: Chloromethyl derivative of 2', 7'-dichlorodihydrofluorescein diacetate
DCF: 2', 7'-Dichlorodihydrofluorescein
DMEM: Dulbecco’s Modified Eagle’s Medium
ECM: Extracellular matrix
FF: Fibroblastic foci
HE staining: Hematoxylin-Eosin staining
IPF: Idiopathic pulmonary fibrosis
LF: Lung fibroblasts
LPS: Lipopolysaccharide
MAP kinase: Mitogen activated protein kinase
NAC: N-acetylcysteine
NOX: NADPH oxidase
PI3K: Phosphoinositide 3-kinase
ROS: Reactive oxygen species
SEM: Standard error of the mean
siRNA: Small interfering RNA
TGF-β: Transforming growth factor-β
WB: Western blotting
αSMA: α-smooth muscle actin
